# Promoting Healthy Eating among Young People—A Review of the Evidence of the Impact of School-Based Interventions

**DOI:** 10.3390/nu12092894

**Published:** 2020-09-22

**Authors:** Abina Chaudhary, František Sudzina, Bent Egberg Mikkelsen

**Affiliations:** 1 Independent Researcher, Kastrupvej 79, 2300 Copenhagen, Denmark; abinachaudhary@yahoo.com; 2 Department of Materials and Production, Faculty of Engineering and Science, Aalborg University, A. C. Meyers Vænge 15, 2450 Copenhagen, Denmark; 3 Department of Systems Analysis, Faculty of Informatics and Statistics, University of Economics, nám. W. Churchilla 1938/4, 130 67 Prague, Czech Republic; 4 Department of Geosciences and Natural Resource Management, Faculty of Science, University of Copenhagen, Rolighedsvej 23, 1958 Frederiksberg C, Denmark; bemi@ign.ku.dk

**Keywords:** school children, food and nutrition, intervention, healthy eating

## Abstract

Intro: Globally, the prevalence of overweight and obesity is increasing among children and younger adults and is associated with unhealthy dietary habits and lack of physical activity. School food is increasingly brought forward as a policy to address the unhealthy eating patterns among young people. Aim: This study investigated the evidence for the effectiveness of school-based food and nutrition interventions on health outcomes by reviewing scientific evidence-based intervention studies amongst children at the international level. Methods: This study was based on a systematic review using the PRISMA guidelines. Three electronic databases were systematically searched, reference lists were screened for studies evaluating school-based food and nutrition interventions that promoted children’s dietary behaviour and health aiming changes in the body composition among children. Articles dating from 2014 to 2019 were selected and reported effects on anthropometry, dietary behaviour, nutritional knowledge, and attitude. Results: The review showed that school-based interventions in general were able to affect attitudes, knowledge, behaviour and anthropometry, but that the design of the intervention affects the size of the effect. In general, food focused interventions taking an environmental approach seemed to be most effective. Conclusions: School-based interventions (including multicomponent interventions) can be an effective and promising means for promoting healthy eating, improving dietary behaviour, attitude and anthropometry among young children. Thus, schools as a system have the potential to make lasting improvements, ensuring healthy school environment around the globe for the betterment of children’s short- and long-term health.

## 1. Introduction

Childhood is one of the critical periods for good health and development in human life [1,2]. During this age, the physiological need for nutrients increases and the consumption of a diet high in nutritional quality is particularly important. Evidence suggests that lifestyle, behaviour patterns and eating habits adopted during this age persist throughout adulthood and can have a significant influence on health and wellbeing in later life [3,4]. Furthermore, the transition from childhood into adolescence is often associated with unhealthy dietary changes. Thus, it is important to establish healthful eating behaviours early in life and specially focus on the childhood transition period. A healthy diet during the primary age of children reduces the risk of immediate nutrition-related health problems of primary concern to school children, namely, obesity, dental caries and lack of physical activity [5,6,7]. Furthermore, young people adopting these healthy habits during childhood are more likely to maintain their health and thus be at reduced risk of chronic ailments in later life [7,8,9]. Thus, healthy behaviours learnt at a young age might be instrumental in reaching the goals of good health and wellbeing of the 2030 Sustainability Agenda which has implications at the global level.

Globally, the prevalence of overweight and obesity rose by 47.1% for children and 27.5% for adults between 1980 and 2013 [10]. A recent WHO (World Health Organization) Commission report [10] stated that if these same trends were to continue, then by 2025, 70 million children are predicted to be affected [11]. Hence, the increased prevalence might negatively affect child and adult morbidity and mortality around the world [12,13]. Worldwide the dietary recommendations for healthy diets recommend the consumption of at least five portions of fruits and vegetables a day, reduced intake of saturated fat and salt and increased consumption of complex carbohydrates and fibres [14]. However, studies show that most children and adolescent do not meet these guidelines [15,16] and, thus, as a result, childhood and adolescent obesity are alarming nearly everywhere [17]. Recent figures show that the prevalence has tripled in many countries, making it the major public health issue in the 21st century [18,19,20,21]. According to WHO [4], 1 in 3 children aged 6–9 were overweight and obese in 2010, up from 1 in 4 children of the same age in 2008.

The increased prevalence of overweight and obesity has fuelled efforts to counteract the development, as seen for instance in the action plan on childhood obesity [17]. Increasingly policy makers have been turning their interest to the school setting as a well-suited arena for the promotion of healthier environments [18]. As a result, schools have been the target of increased attention from the research community to develop interventions and to examine the school environment to promote healthful behaviours including healthy eating habits.

Globally, interventions in the school environment to promote healthier nutrition among young people have received considerable attention from researchers over the past years. But there is far from a consensus on what are the most effective ways to make the most out of schools’ potential to contribute to better health through food-based actions. Is it the environment that makes a difference? Is it the education or is it the overall attention given to food and eating that plays the biggest role? School food and nutrition intervention strategies have witnessed a gradual change from knowledge orientation to behavioural orientation [22] and from a focus on the individual to the food environment. Research evidence has shown that adequate nutrition knowledge and positive attitudes towards nutrition do not necessarily translate to good dietary practices. Similarly, research has shown that the food environment plays a far bigger role in behaviour than originally believed [23,24].

School-based interventions can a priori be considered as an effective method for promoting better eating at the population level. Schools reach a large number of participants across diverse ethnic groups. It not only reaches children, but school staffs, family members as well as community members [8,25]. Schools can be considered a protected place where certain rules apply and where policies of public priority can be deployed relatively easily. In addition, schools are professional spaces in which learning and formation is at the heart of activities and guided by a skilled and professional staff. Schools, as such, represent a powerful social environment that hold the potential to promote and provide healthy nutrition and education. Besides the potential to create health and healthy behaviours, good nutrition at school has, according to more studies, the potential to add to educational outcomes and academic performance [26,27,28]. 

However, taking the growth in research studies and papers in the field into account, it is difficult for both the research community and for policy makers to stay up to date on how successful school-based interventions have been in improving dietary behaviours, nutritional knowledge and anthropometry among children. Also, the knowledge and insights into how it is possible to intervene in the different corners of the school food environment has developed which obviously has influenced over recent decades how programs and interventions can be designed. It has also become clear that food at school is more than just the food taken but includes curricular and school policy components. The findings from school-based studies on the relationship between school, family as well as community-based interventions and health impact suggest that health impacts are dependent on the context in which they have been carried out as well as the methodology. Thus, an updated overview as well as a more detailed analysis of initiatives is needed in order to develop our understanding of the nature of the mechanisms through which the school can contribute to the shaping of healthier dietary behaviour among children and adolescents before more precise policy instruments can be developed. Our study attempted to fill the need for better insight into which of the many intervention components works best. It attempted to look at school food and nutrition interventions reported in the literature that have been looking at healthy eating programmes, projects, interventions or initiatives.

School-based interventions in the Western world are traditionally targeted at addressing obesity and over-nutrition, but school food interventions are also addressing under nutrition and, as such, their role in a double burden of disease perspective should not be underestimated. Many studies have reported on micronutrient malnutrition among school-aged children in developing countries (for instance [29,30,31]) but it has also been reported in the context of developed countries [32]. Against this backdrop, the aim of this study was to provide an analysis of the evidence of the effectiveness of school-based food interventions by reviewing recent scientific, evidence-based intervention studies on healthy eating promotion at school. The specific objectives of the study were to identify which interventions had an effect on primary outcomes, such as BMI, or on secondary outcomes such as dietary behaviour, nutritional knowledge and attitude.

## 2. Materials and Methods

The functional unit of the review were healthy eating programmes, projects or initiatives that have been performed using the school as a setting. We included only programmes, projects or initiatives that were studied in a research context, in the sense that they were planned by researchers, carried out under controlled settings using a research protocol, and reported in the literature. School-based programmes, projects, interventions or initiatives are, per definition, cluster samples where a number of schools first were chosen for intervention followed by performing an outcome measurement before and after the intervention and, in most cases, also in one or more control schools. The outcome measurement in the studies reviewed was performed on a sample of students that was drawn from each school (cluster).For this, the systematic review and meta-analysis (PRISMA) guidelines and the standardised quality assessment tool “effective public health practice project (EPHPP) quality assessment tool for quantitative studies” were used for analysing the quality assessment of the included studies [33]. This EPHPP instrument can be used to assess the quality of quantitative studies with a variety of study designs.

### 2.1. Literature Search

The literature review involved searches in PubMed, Web of Science and Cochrane Library database. The search strategy was designed to be inclusive and focused on three key elements: population (e.g., children); intervention (e.g., school-based); outcome (e.g., diet and nutrition, knowledge, attitude and anthropometrics). The search terms used in PubMed database were: “effectiveness of school food AND nutrition AND primary school children”, “effectiveness of school food AND nutrition AND interventions OR programs AND among primary school children AND increase healthy consumption”, “primary school children and education and food interventions”, “Effectiveness of school-based food interventions among primary school”, “effectiveness of school-based nutrition and food interventions”, “primary school interventions and its effectiveness”, and “obesity prevention intervention among Primary schools”. Search terms such as: “effectiveness of school-based food interventions among primary school”, “effectiveness of school based food and nutrition interventions”, “primary school interventions and its effectiveness” and “obesity prevention interventions”, were used in the Web of Science database. Lastly, search terms such as: “nutrition interventions in primary schools” and “Nutrition education interventions in school” were used in the Cochrane Library database to find the articles. In addition, reference lists of all retrieved articles and review articles [34] were screened for potentially eligible articles. The search strategy was initially developed in PubMed and adapted for use in other databases. In addition, snowballing of the reference list of the selected articles was conducted.

### 2.2. Inclusion Criteria

Studies selected for the inclusion were studies which investigated the effectiveness of a school-based interventions targeting food and nutrition behaviour, healthy eating and nutrition education as a primary focus during the intervention. Also, to be included in this review, only articles from 2014 to 2019 were selected and of those inclusion criteria included articles targeting primary school children aged between 5 and 14 years. Participants included both boys and girls without considering their socio-economic background. Study design included randomized controlled trial “RCT”, cluster randomized controlled trial “RCCT”, controlled trial “CT”, pre-test/post-test with and without control “PP”, experimental design “Quasi”. Studies which did not meet the intervention components/exposures, such as information and teaching (mostly for the target group and parents were additional), family focus on social support and food focus (which mainly focuses on the availability of free foods including food availability from school gardening), were excluded. Systematic review papers and studies written in different language except for English were excluded as well. Studies which met the intervention criteria but had after school programs were excluded.

### 2.3. Age Range

Since the review covers a broad range of different countries and since school systems are quite different, the sampling principle had to include some simplification and standardisation. The goal of the review was to cover elementary (primary) and secondary education and, as a result, the age range of 5–14 was chosen to be the best fit, although it should be noted that secondary education in some countries also covers those 15–18 years of age. In most countries, elementary education/primary education is the first—and normally obligatory—phase of formal education. It begins at approximately age 5 to 7 and ends at about age 11 to 13 and in some countries 14. In the United Kingdom and some other countries, the term primary is used instead of elementary. In the United States the term primary refers to only the first three years of elementary education, i.e., grades 1 to 3. Elementary education is, in most countries, preceded by some kind of kindergarten/preschool for children aged 3 to 5 or 6 and normally followed by secondary education.

### 2.4. Assessment of Study Eligibility

For the selection of the relevant studies, all the titles and abstracts generated from the searches were examined. The articles were rejected on initial screening if the title and abstract did not meet the inclusion criteria or met the exclusion criteria. If abstracts did not provide enough exclusion information or were not available, then the full text was obtained for evaluation. The evaluation of full text was done to refine the results using the aforementioned inclusion and exclusion criteria. Thus, those studies that met predefined inclusion criteria were selected for this study.

### 2.5. Analytical Approach

The first step of data collection was aimed at organizing all studies with their key information. In the second step, we created coded columns. A coded column served as a basis for being able to do further statistical analysis. In other words, in a coded column we added a new construct not originally found in the papers as a kind of dummy variable that standardized otherwise non-standardized information, allowing us to treat otherwise un-calculable data statistically. For the impact columns, we used the following approach to construct codes where impacts where put on a 1–4-point Likert scale with 1 being “ineffective”, 2 “partially effective”, 3 “effective” and 4 “very effective”.

For the design column, the following approach was adopted as illustrated in the Table 1. Quasi experimental/pre–post studies were labelled QED and were considered to always include a baseline and follow-up outcome measurement. As the simplest design with no comparison but just a pre/post study of the same group, we constructed a power column and assigned 1 to this for a QED design. For the controlled trial (CT), we assigned the power 2. A controlled trial is the same as QED but with a comparison/control in which no interventions are made and with no randomization. We considered a study to be of that kind if some kind of controls were made which could be, for instance, matching. All CTs in our study included 2 types of comparisons: pre and post (baseline and follow-up) as well as a comparison between intervention/no intervention. For the RCT/RCCT—a trial that is controlled through the randomization—we assigned the power 3. This “top of hierarchy” design includes the case (intervention) and a control (no intervention) and normally two types of comparisons (pre and post) as well as an intervention/no intervention. For the context of this study, we did not differentiate between RCTs and RCCTs. The latter is sometimes used to stress the fact that the school (or the class) is the sampling unit from which the subjects are recruited. But since in the context of schools RCCT is simply a variation of RCT, we coded them in the same class of power. We simply assumed that when authors spoke about an RCT, they in fact meant an RCCT since they could not have been sampling subjects without using the school as the unit.

Codes and categorization were used to standardize the information found in the papers for our statistical analysis. Categorisation of the age/class level, such as EA—Early age, EML—Early middle late, EL—Early late, was used. 

For the intervention components (“what was done”) we translated all studies into three columns: information and teaching, family and social support and environmental components, food provision and availability. The latter was further expanded into three columns labelled as: focus on and provisioning of F & V; free food availability through school gardening and availability of food and healthier food environment. Our inclusion criteria were that studies should contain at least one of these components. For the environmental component—food provision and availability intervention components—we identified 2 distinct types: either a broad healthier eating focus or a narrow and more targeted fruit and vegetable focus. After the coding, we started to ask questions about the data. Most importantly, we were interested in knowing whether there existed a relationship between “what was done” and “what was the impact”. In other words, we were interested in knowing more whether there was a pattern in the way the studies intervened and the outcomes.

### 2.6. Queries Made

We performed queries for each intervention component (the independent variable in columns K, L and M) for each single outcome measure.

Is there a relationship between age and outcome? We used the coded column (EA, EML, etc.) to study that relationship.

In addition, we made queries regarding the relationship among study designs. For instance, would the duration of studies influence whether an effect could be found or not? Would more powerful designs result in more impact?

Furthermore, we made queries on the relationship between one intervention and a multi-interventional component and their effect on the outcome measure. Also, the queries on target groups were made. Codes such as S and NS (refer Table 4) in the column were used to study the relationship. In our analysis a distinction was made between “standard” and “extreme” (special cases). From the reviewed papers, it was clear that some studies put little emphasis on the school selected. We classified those as standard (S). However, a few papers used a stratification approach and case/cluster selection that can be classified as an “extreme” or non-standard case. We coded these as non-standard (NS). For instance, studies could be targeted to include only refugees or subjects of low socio-economic status. It can be speculated that being a “special case” or extreme case could have an influence. As a result, we reserved a code for these cases, although it became clear that they represented only a minority.

In our study, availability plays a central role, since it is used in many food-at-school intervention studies. Availability signals that food is “pushed” as opposed to being used in the “pull” mode, where individuals are expected to request food in the sense that is the behaviour of the individual that becomes the driving force rather than the “out thereness”. Availability is in most studies used in combination with the idea of a food environment. The literature shows that availability can be of two types. One is when food is made available for the individual to take where visibility, salience, product placement, etc., are used as factors. The other type of availability is when it is made free and the individual as a result does not have to pay. Free availability has been studied extensively in intervention studies but for obvious reason it is difficult to implement “post-study” since there needs to be a permanent financing present. The only exceptions to this are the collective meal models found in countries such as Sweden, Finland, Estonia and Brazil as well as in the EU scheme where the EU subsidizes the fruit.

Study design and other characteristics are provided in Table 2, and their findings are provided in Table 3.

The information from abstracts were organized in a table with the following information:

Column A: Authors. The column lists the researchers/authors conducting the study.

Column B: Year. The column shows the year of the publication of the article.

Column C: Title/Reference. The column lists the title of the article.

Column D: Main aim. The column lists the main aim presented by authors in the abstract of each article.

Column E: Main aim in brief. This column is a constructed variable that refers to the main aim of each study. The idea was to give in brief the study idea and which outcome measures was focused on in the study.

Column F: Program name. The column gives the name of the project, program or intervention reported in in the article.

Column G: Location and Country. The column lists the specific place or location where the study was performed.

Column H: Study design. The column shows research design of the study according to authors.

Column I: Study design coded. This column is a constructed variable to capture the research design of the study and used to make an analysis of power possible, see Column J.

Column J: Power. The column was constructed to express the strength of the design. It is a dummy variable that was assigned a numerical value that allowed for a quantitative analytical approach.

Column K, L and M: Intervention components. The column shows which intervention components that was used in the study. We used a model that categorizes components into three different mechanisms of influence: cognitive (K), environmental (L, M, N) and social (O).

The environmental component includes actions where availability of meals—or fruit and vegetable (F & V)—were increased. Either through passive provision (F & V and meals) or through active participation such as gardening. The social category included actions where families and/or peers were actively influencing the participants. The cognitive category included teaching and learning.

Column L: Environmental/food focus on F & V. In this column, interventions which were targeted towards fruits and vegetables were flagged. This includes interventions whose focus was providing cooking lessons and maintaining healthy cafeterias during the intervention periods. Also, maintaining healthy cafeteria here refers to school canteens providing healthy options to its menu where children’s while buying food have healthier options to choose. 

Column M: Environmental/food focus on increasing availability through school gardening. In this column, interventions which provided free foods among participants through gardening within the school were listed.

Column N: Environmental/food interventions focused on healthy meal availability. Interventions which provided healthy meals, breakfast, snacks during the school hours and distributed fresh fruits among the participants were listed in this column.

Column O: Family/social support. In this column interventions that included social components were flagged. These interventions included peer and family influence mechanisms.

Column P: Age. The column lists the age of the targeted groups of the intervention expressed in years according to the primary article data provided by authors.

Column Q: Age construct EA. This column shows a constructed variable for the age categorization based on the primary data given by authors. The constructed code was made to make statistical analyses possible. The construct Early Age (EA) was assigned if intervention were carried out in early school.

Column R: Age construct EML. This column shows a constructed variable for the age categorization based on the primary data given by authors. The code Early Middle Late (EML) was assigned if intervention was targeted all age groups.

Column S: Age construct EL. This column shows a constructed variable for the age categorization based on the primary data given by authors. The code EL refers to Early late and was assigned if the intervention was targeted early and early and late school.

Column T: Sample size. The number of young people enrolled in the intervention was listed in this column.

Column U: Time duration. This column shows the length of the intervention expressed in months. It is a constructed variable based on the primary data given by authors and was made to standardize duration and make it ready for cross study analysis.

**Figure 1:**
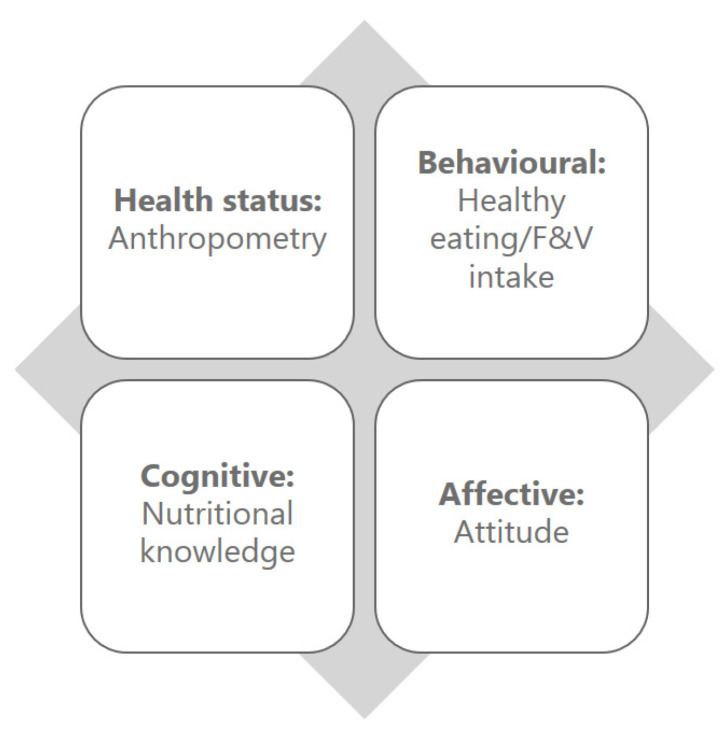
Outcome measures model. The figure illustrates the four types of outcome measures found in the interventions.

Columns V, W, X, Y: Outcome measures. In Columns T, U, V, W, the outcome measures named as Anthropometry, HE/FV (healthy eating fruits and vegetables), Nutritional knowledge, and Attitude, respectively, were listed according to our outcome model shown in Figure 1. Only a few include all outcome measures, but all studies included at least one of them.

Columns X, AA, AB, AC: Effectiveness. The effectiveness as measured by the outcomes measured are listed in this column. Each outcome measure was rated using a Likert scale from 0–4. The effectiveness of outcome measures among participants as measured by the measures in our model (Figure 1): attitude, anthropometry, HE/FV, nutritional knowledge and attitude were listed in the Columns X, Y, Z, AA, respectively.

Column AD: Target group. This column provides information on the target group of interventions such as information on grades of subjects and municipalities.

Columns AE, AF: Target group. This column is a constructed variable created to capture if the intervention had a special ethnic or socio-economic focus. Columns AC and AD consisted of coded target group named as Standard (S) and Non-Standard (NS). The “NS” here represents the target group either from refugees or immigrants or lower socio-economic classes.

Column AG: Keywords. This column lists the keywords found in the interventions.

Ordinary least squares regression was applied in this study; specifically, we used the linear regression function in IBM SPSS 22. We opted for a multi-variate approach; i.e., multiple linear regression was used. Anthropometry, behaviour (healthy eating and food focus), attitude and nutritional knowledge were used as dependent variables. In order to better account for control variables, such as sample size and study length, a dummy variable was introduced for study length of one year and more; and a logarithm of the sample size was used instead of the actual sample size to eliminate scaling effects. We grouped countries by continents (while splitting Europe into North and South as there were enough studies and no countries in between) and introduced related dummy variables. The remaining variables were used as independent variables without any additional manipulations.

Since the aim was to create models consisting only of independent variables that significantly influence the dependent variables, we used the backwards function. Because there were too many independent variables for the backwards function for the attitude model (with only eight observations), the stepwise function was used instead.

Information and teaching was present in all but one study. Free food was found only in two studies and focus on fruit and vegetables in three studies. Therefore, it is not surprising that neither of the three variables were found to be significant in any of the models.

### 2.7. Study Sample

**Figure 2:**
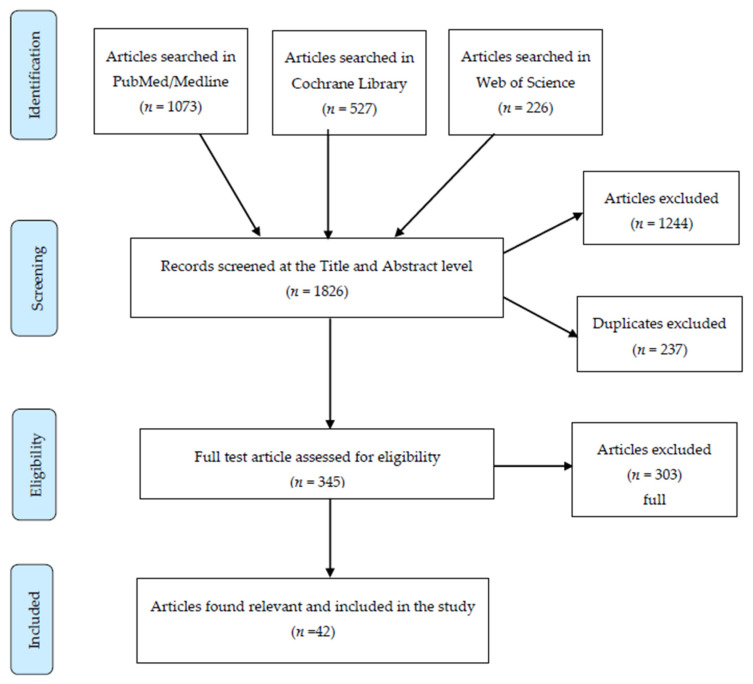
Review flow chart. The figure shows the progress of the literature review process following the PRISMA 2009 approach.

The search strategy resulted in 1826 titles which were screened for duplicates and potential relevance. After this initial screening, 345 titles and abstracts were assessed against the inclusion and exclusion criteria. Articles that studied school interventions after school hours were excluded. In addition, articles which studied interventions among children in out of school context such as at community level were excluded. The justification is that both “after school” and “out of school” since can be regarded as non-typical school environments. We aimed to study the “school” as an artefact that can be considered as a “standard” across countries despite some national differences. For both “after school” and “out of school”, we argue that there are considerable differences among countries and that an inclusion of such studies would negatively influence our analytical approach. In total, 42 articles were identified as relevant and full papers were obtained as the final sample. Figure 2 below illustrates the search terms and selection process of articles.

### 2.8. Intervention Study Characteristics

For all 43 items in our sample, Table 2 provides the information about the study, intervention methodologies, characteristics strategies, etc. In our extract of studies, the sample size ranged from 65-2997 subjects/participants, and the intervention duration ranged from 1 and half month to 36 months. The systematic review locations identified by the author were: 26 from Europe [21,36,38,39,40,44,46,49,52,54,57,58,63,64,65,66,67,68,69,70,71,72,73,74,75], six from Asia [35,42,48,59,60,62], 10 from America [37,41,43,45,47,50,51,53,55,61] and one from Africa [56]. We categorized all interventions according to their intervention components. To this end, we had constructed three classes: Information and Teaching, Food Focus and Family/Social support as illustrated. The interventions characteristics of each included study are shown in Table 2.

Of the total study sample, the majority of studies (*n* = 41) involved “Information and Teaching” components consisting mainly of classroom-based activities (e.g., an adapted curriculum and distribution of educational materials, health and nutrition education program). Another 12 studies along with “Information and Teaching” involved a food focus and availability component. These food and availability components which consisted mainly of supervised school gardening, environmental modifications to stimulate a more healthful diet, such as increased availability and accessibility of healthy foods, distributions free food programmes, school provided free breakfast, school lunch modifications and incentives. Only two studies combined all the three intervention components of this study. Family/social support intervention was clearly focused on in nine study. In other studies, even though their interventions were not primarily or secondarily focused on family/social support component, they indirectly acknowledged the importance of parents and included them in their studies.

All of the reviewed studies included intervention components that were delivered in school settings and within school hours. Our sample showed that consumption of fruit and vegetables was the most used intervention component and was include in more than half of the interventions. Most studies were designed and carried in a way where a research assistant was trained by senior researchers/co-authors to ensure that each members of the research team followed same procedures for data collection. Since all studies were “in situ” studies included a close researcher/school staff cooperation component. In most of the listed studies, teachers being the responsible person to implement the interventions were trained beforehand.

### 2.9. Types of Interventions

Table 2 shows an overview of the programmes and their intervention components. From the table, it can be seen that studies differed according to how broadly they intervened. Some studies have included a narrow intervention (i.e., only one intervention components which targeted behavioural components), whereas others included multicomponent approaches where all three intervention components were used in the study.

## 3. Results

Finding the right approach to intervening for healthier eating at school is a major challenge. In other words, which interventions create which impacts and how should the public best invest in new policies, strategies, and practices at school if long term health is the intended end point?

The purpose of this review was to compile the evidence regarding the effectiveness of successful school-based interventions in improving dietary behaviours, nutritional knowledge, attitudes and anthropometry among children. The analysis of the data showed a number of relationships between outcome effect and a number of other characteristics of the intervention (i.e., age, location/region, intervention type, duration). Descriptive statistics are provided in Table 4.

The linear regression models carried out for each intervention component is added in the text and the tables have been referred to each associated result. Out of 42 studies, 36 studies reported the outcome on HE/FV behaviour scale while anthropometry and attitude impacts were observed in 18 and six studies, respectively. The item one of the results in this article presents the most general finding from the literature review, item two describes the variable found significant in two cases, while the remaining variables were significant in once case each. Additionally, item four, five and six are related “design” phenomena effects in the sense that they are not related to intervention components but to the study was designed your study. The rest is related to (intervention components rather than designs. In Table 5, the outcome measures for which an effect could be seen has been listed. The linear regression model describing what influences the attitude is provided in Table 6.

With regards to the explanatory power of the model, *R*^2^ = 0.789, *R*^2^ adj. = 0.719, and significance = 0.009.

The linear regression model describing what influences the anthropometry is provided in Table 6.

With regards to the explanatory power of the model, *R*^2^ = 0.683, *R*^2^ adj. = 0.586, and significance = 0.003.

The linear regression model describing what influences the behaviour is provided in Table 7.

With regards to the explanatory power of the model, *R*^2^ = 0.121, *R*^2^ adj. = 0.096, and significance = 0.037.

An alternative linear regression model describing what influences the behaviour is provided in Table 8.

With regards to the explanatory power of the model, *R*^2^ = 0.449, *R*^2^ adj. = 0.432, and significance < 0.001.

### 3.1. School-Based Interventions in General Create Impact

Looking across the whole study sample, it can be seen that in general the interventions created an impact in one or more ways either on knowledge, intentions, eating habits and/or anthropometry. In other words, it was hard to find studies that created no impact. This finding adds to the body of evidence that suggests that food-based interventions are a well-suited and effective policy tool when it comes to promoting healthier eating among young people.

### 3.2. Family Support Affects Healthier Eating Behaviour and Attitude

Out of all the included studies, nine studies focused on family support as an intervention component. But out of those, our analysis showed that the family involvement was impactful among participants when it comes to promoting healthier food choices. Parents being influencers and role models in the family in these studies seemed to help to influence children’s dietary habits. Studies which involved participants’ parents in the intervention and provided them with nutritional knowledge and healthy cooking skills (i.e., knowledge about the importance of healthy food and nutrition during the early age of their children), seemed to be able to help young people prepare more healthy and nutritious food at home. As studies showed, this seemed to increase children’s intentions towards eating more fruits and vegetables and eventually resulted in consumption of more healthy foods. However, this did not seem to be the case for all ages. Intention to eat more fruits and vegetables was seen among early age participants (EA) either alone or with family support. It should be noted that the regression models did not include interactions, since the number of analysed studies was only ~40. It was not possible to include age as a continuous variable in the models because (as it can be seen in Table 5) age was a range, and sometimes even a wide range, e.g., 8–11 or 4–11. Family support increases the outcome measure by approximately 1 in both cases. Please refer to Table 5 and Table 7 for detailed linear regression model used for attitude and behaviour.

### 3.3. Interventions Done in Northern Europe (7 Studies) Had a Smaller Impact on Behaviour than the Studies Conducted in the Rest of the World (22 Studies)

The results from the models which was created to measure the efficiency of HE/FV highlighted the fact that HE/FV scale depends only on region where the intervention was done. The behaviour outcome for Northern Europe was on average 1.5 while the average for the rest was 3.2 (please refer to Table 8).

### 3.4. Effect of Anthropometry Measures Increases with Study Power

The results suggested that the design of the study plays a role when it comes to be able to show impact of interventions. From the findings, it was clear that the anthropometry measured among the participants were increasing with the power of the study. That is, the stronger the design the greater the likelihood of being able to measure impact on anthropometric outcomes—a unit increase in the design power is associated with an outcome increase of approximately 1.5 (please refer to Table 6). To examine the influence of study design we used the score that was constructed for the purpose (please refer to Table 1). This score assigns a higher power to randomized designs than non-randomized ones.

### 3.5. Study Duration Impacts Anthropometric Outcomes

It was also clear that the intervention duration does have impact on the outcome, i.e., the longer the duration better the anthropometric results among the children. Interventions that lasted a year or more, had the outcome measure on average almost one unit higher than shorter studies (please refer to Table 6).

### 3.6. Larger Samples Impacts Anthropometry Measures

Results showed that anthropometric outcome decreased within the sample size. Increasing the sample size by a factor of 10, from approximately 100 to 1000, decreased the outcome measure by almost 2.5 (please refer to Table 6). Thus, bigger the sample size a reverse effect on outcome was obtained. The studies whose intervention was done for long period of time (i.e., couple of months or year and among small participants) were found to be effective in the outcome. It might be the case that it was hard to administer the same thing to large sample size post intervention and thus could have decreased the anthropometry outcome among the participants.

### 3.7. Food Availability Interventions Influence Anthropometric Outcomes

Our analyses showed that a food focus, specifically healthy meal availability had an impact on the children’s anthropometric outcomes—increasing it by almost 3.5 on average (please refer to Table 6).

### 3.8. Interventions among Younger Students Influence Attitude Among Participants

Results showed that the younger the study subjects were, the more influence interventions had on attitudes (the outcome was on average 0.75 higher than for other age groups). Thus, the result suggests that the participants’ attitude increases when they are in their early age (EA) i.e., 4–7 years old. Furthermore, results suggest that increased family support associated with participants’ attitude towards healthy eating helps in changing the behaviour among them. Early age (EA) and family support seemed to impact positively both alone and together. Meaning that the intervention had positive impacts on participants (i.e., EA participants) attitudes towards healthy eating either with the involvement of their family support or without the involvement of family support. Please refer to Table 5 for detail linear regression model for attitude.

### 3.9. No Effect of School Based Interventions on Nutritional Knowledge

Findings showed that nutritional knowledge among participants (i.e., of all age group) does not depend on school-based interventions. Thus, none of the collected variables have influences on nutritional knowledge.

## 4. Discussion

### 4.1. Discussion of Results of This Review in Relation to Others

In the discussion we aim to relate our findings with what has been found in previous studies, discuss our methodological approach and reflect on what are the policy implications. Since the discussion on how to counteract the unhealthy eating pattern and the worrying increase in nutrition related disorders among young people is attracting much attention and since the discussion on how the school could contribute we aim to give policy makers and practitioners an up to date insight into the potentials of the school to act as a hub for promotion of healthier eating and provide inspiration for the development of new types of school-based interventions and strategies.

The huge interest in using the infrastructure of the school to initiate and promote healthier eating among young people has resulted in a large number of interventions studies over the past decades. This research interest per definition as the same time creates a need for syntheses of the findings in order to make them feed into the public health and school policy cycle and to “send the results to work”. Taken the huge investment that better food at school strategies at school will cost for states it is worth appreciating that the Evidence-Informs-Policy pathway seems to be working. At the same time the conceptual approaches and the understanding of what intervention components might work better than others, which age groups might benefit the most etc. as developed considerably which again adds to the rationale for synthesis of intervention study findings. Most recent reviews by Julie et al. [76], Noguera el al. [77], Evans et al. [78], Cauwenberghe et al. [34] and Brown et al. [79] has created a time gap of almost five years. Covering the last five years of research our review makes a needed contribution and in addition we argue it makes a needed contribution to a standardization and conceptualization of both sampling and intervention design methodologies.

Overall, the findings from this review suggest that school-based interventions that include intervention components such as information and teaching, food focus and family support are effective in improving the HE/FV, anthropometric measurements and attitude towards healthy dietary behaviour among the participants. On the other hand, nutritional knowledge among participants did not seem to be influenced much by any of the intervention components used.

Impacts on HE/FV behaviours were observed, but mostly among early age children revealing a distinct age pattern in the findings. Thus, age was seen as a significant factor in determining effectiveness in several study [35,37,39,42]. Impact was greater on young children in the 4–7 year old age range, suggesting that dietary influences may vary with age.

Multicomponent approaches that includes good quality instruction and programs, a supportive social environment both at school and home, family support has been effective in addressing childhood related diseases through focusing on diet and physical activity. Most of the studies in this review implemented with combination of school staff and intervention specialists provide evidence for the effectiveness of the program. Thus, evidence supports that family involvement and nutrition education curriculum delivered by the teacher under supervision of intervention specialists can alter the intake of fruit and vegetables while impacting positively on anthropometric measurements. Teacher led interventions have been effective and can be the most sustainable approach for long term impact of the program. The same conclusion was found in a review done in investigating the effectiveness of school-based interventions in Europe which provided the effectiveness of multicomponent intervention promoting a healthy diet in school aged children in Europe [34].Studies with a food focus in their intervention approaches showed significant improvements in BMI [35,54,58]. Significant improvements in BMI here refers to the studies whose probability value was less or equal to 0.05. This means that the interventions in that case showed reduction in body mass of participants. We looked at studies whose aim was to focus on interventions of obesity prevention or reduction among primary school children’s. Thus, search term such as: “obesity prevention intervention among primary schools”, was used as explained in the methods section. When performing the search for school-based interventions we did not encounter any studies that were focusing on underweight. Making the options for healthy choices of food in the school cafeterias and having the option of free food from the school gardens decreases the sugar sweetened beverages and junk options among the children’s and thus resulting in improvements in BMI. This review evidence further highlights that duration of the intervention, i.e., a year or more has an impact on anthropometric measurements. This is in contrast to reviews of Julie et al. [76] and Cauwenberghe et al. [34] review that found that making the better options of food choices and duration of the studies were effective in reducing the sedentary behaviour and noting improvements in BMI. This study also found that larger sample sizes reverse the outcome of anthropometric measurements (i.e., sample size negatively influences the outcome). This might be the case because it might be harder to administer the same thing to more individual. Thus, more studies are needed to examine the effects of bigger sample sizes.

Our study is far from being the first to create overview of the large number of studies that are studying interventions that can promote healthier eating habits and that can counteract the worrying increase in obesity and overweight among young people the general. The huge interest is reflected in the number of studies trying to assess the impact and effectiveness of school-based interventions as well as in the number of reviews aiming to synthesize the findings from the growing body of evidence of the effect of school-based food interventions into actionable school food policies. Our study adds to this body of knowledge and fills a gap since our study looks at the most recent studies.

Comparing our review with others we find that the majority of the studies on school food-based interventions have been conducted in high income countries. This is also the case in our study and this fact is important to keep in mind since it introduces a bias in the insight created from school food effectiveness reviews. It is also important to keep in mind that studies—and as a result also reviews-covers different types of school food cultures. These cultures can roughly be divided in collective, semi collective and non-collective types. In the collective type found in countries such as Sweden, Finland, Estonia and Brazil school food provision is an integrated—and mainly free—part of the school day. In semi-collective approaches food is in most cases traditionally a part of what is offered at school, but due to payment. In the non-collective approach found in countries such as Denmark, Norway and the Netherlands there is little infrastructure and tradition for school organized foodservice. In this approach parents organized lunch boxes as well as competitive foods traditionally play a bigger role.

A further important note to make is the distinction between narrow F & V approaches and broader healthier eating intervention approaches. This classification can also be seen in previous studies and in more recent reviews. The first type of interventions that follow the six-a-day tradition that to some extent has been fuelled by the European School Fruit program introduced by the EU in 2009 was reviewed by Noguera et al. [77] and by Evans et al. [78]. In a study by Noguera el al. [77] a meta-analysis on F&V interventions was done but limited to educational interventions in the sense that it only looked at computer-based interventions and covering mostly European research. The study showed that this targeted but narrowed approach was effective in increasing FV consumption but that broader multicomponent types of interventions including free/subsidized FV interventions were not effective. In the review paper from 2012 by Evans et al. [78] examined studies done in United Kingdom, United States, Canada, Denmark, New Zealand, Norway and the Netherlands. Evans and co-workers [78] found that school-based interventions were able to moderately improve fruit intake but that they had only minimal impact on vegetable intake. These reviews and previous ones generally conclude that F&V targeted interventions are able to improve young people’s eating patterns towards higher intake of fruit.

In the category of reviews taking a broader approach to healthier lifestyle promotion we find studies and reviews that looks at promotion of healthier eating in general—and that in some cases include physical activity. A review by Julie et al. [76] covered studies from United States, United Kingdom, Australia, Spain and the Netherlands. This review also included physical activity as part of broader school-based obesity prevention interventions. In particular, interventions should focus on extending physical education classes, incorporating activity breaks, and reducing sedentary behaviours to improve anthropometric measures. Julie et al. concluded that interventions taking a broader approach should include employing a combination of school staff and intervention specialists to implement programs; that they should include psychosocial/psychoeducational components; involve peer leaders; use incentives to increase fruit and vegetable consumption and should involve family. In a study by Cauwenberghe et al. [34] intervention studies done in a European union studies were reviewed. This review—as our study do—made an age distinction in the sense that a categorization was done between children and adolescents. Among children the authors found a strong evidence of effect for multicomponent interventions on fruit and vegetable intake. For educational type of interventions Cauwenberghe et al. [34] found limited evidence of effect as found when looking at behaviour and fruit and vegetable intakes. The study found limited evidence on effectiveness of interventions that specifically targeted children from lower socio-economic status groups. For adolescents Cauwenberghe et al. [34] found moderate evidence of effect was found for educational interventions on behaviour and limited evidence of effect for multicomponent programmes on behaviour. In the same way as our review authors distinguished between behaviour and anthropometrics and found that effects on anthropometrics were often not measured in their sample. Therefore, evidence was lacking and resulted in inconclusive evidence. Cauwenberghe et al. [34] concluded that there was evidence was found for the effectiveness of especially multicomponent interventions promoting a healthy diet but that evidence for effectiveness on anthropometrical obesity-related measures was lacking. In a review by Brown et al. [79] studies mostly from Europe but also covering United States, New Zealand, Canada and Chile it was found that intervention components most likely to influence BMI positively included increased physical activity, decreased sugar sweetened beverages intake, and increased fruit intake.

Our review adds to the increasing support for the idea that school should play a role in promoting healthier eating habits among young people. As such the school can be seen as an important actor when it comes to the promotion of human rights. In particular; the right to adequate food, the right to the highest attainable standard of health and right to the education, school plays an integral part which has also been highlighted in the “United Nations System Standing Committee on Nutrition” new statement for school-based and nutrition interventions [25]. Furthermore, Mikkelsen and colleagues [80] in their study have also suggested the fact that the international framework of human rights should invoke its strategies, policies, and regulations in the context of school and that national, regional, and local level actors has important roles to play. Additionally, they have highlighted that ensuring healthy eating in school environment can be a good investment in children short- and long-term health and education achievements. Thus, schools, as a system have the potential to make lasting improvements in students nutrition both in terms of quality and quantity and simultaneously contribute to realization of human rights around the globe [25].

### 4.2. Discussion of Methods

#### Strengths and Limitations

All attempts to reduce complexity of research studies in a research field suffers from in built weaknesses. Standardising the work of others in attempts to make generalizations is always difficult. As per definition a review includes attempts to standardize its study material in order to create an overview of “what works” and what “this that works” depends on. For obvious reasons research protocols depends very much on the context of the study: What is doable in one study setting on one country might not work on other settings. Additionally, reporting procedures vary among authors. The aim of a review is to standardize this heterogeneity to something that is homogenous and computable. So, in our case our constructs represent an attempt to make different studies with similar but slightly different approaches and methodologies comparable by making them computable. This has obviously some disadvantages.

Another limitation is that our review restricted itself to cover only published English language articles. Therefore, publication bias cannot be excluded, as it is possible that the inclusion of unpublished articles written in other languages than English will have affected the results of this review. Second, most of the studies included in the present were carried out in countries from Southern and Northern parts of Europe. This raises questions about the generalisability of these results to other countries in Europe, especially because contextual variables were often lacking in the included studies. And the same questions about the generalisability could be raise in other parts of the world i.e., in Latin America, North America, Asia and Africa, as very few studies were reported from this part of the world.

On the other hand, large dropouts were reported in many listed studies and the study follow up were reported in few studies and was for short time period. Among these studies which did follow up, was right after the end of the intervention period and thus this could have affected the effectiveness among this study outcomes. Long-term follows-up post-interventions would help to study the retention of behaviour change and effect on the body composition among the participants. Thus, long terms studies post interventions are needed to draw the conclusion about the sustainability of an intervention. Additionally, in future studies to improve the quality of the evidence of effectiveness in this kind of interventions, studies with high quality, rigorous design, appropriate sample size, post interventions long term follow up, assessment of implementation issues and cost effectiveness of the intervention should be executed.

On the strength side the standardisation approach helps to find patterns and to create overview of a large material within a given field of research. The strength of this study is that it provides a broad up to date overview of what is known about the relationship between school-based intervention and policies and healthy eating outcomes among children and that it contributes to the deeper understanding of the fact that current research findings are quite limited. This is among the very few recent reviews which evaluated the effect of school-based food at nutrition interventions among children only. A systematic review approach of this study attempted efficiently to integrate existing information and provide data for researchers’ rationale in the decision making of future research. Furthermore, the applied explicit methods used in this limited bias and, contributed to improved reliability and accuracy of drawn conclusions. Other advantages are that this study looks specifically at the evidence available in Northern and Southern Europe. Statistical analyses of pooled data have facilitated a more through synthesis of the result is one of the biggest strengths of this study.

### 4.3. Policy Implications

The evidence of the impact of school intervention derived from our review suggests several topics to be dealt with in future research not only in Europe but also the other part of the world. First, this review highlights the need for researchers to recognize the importance of further investigations on the measures of anthropometrics, nutritional knowledge, and attitude. Among these 42 studies carried out in different regions very few looked upon the effects on participants’ attitudes and anthropometrics measures. And of those showed positive impact if family support was provided, if started at early age and lastly if food focus was part of the intervention. Additionally, most of the included studies were not aiming to contribute to obesity prevention. Thus, it is highly recommendable that there is urgent need for more studies to be done that includes more measures of efficiency of participants’ attitude towards the healthy behaviour and healthy lifestyle and measures for anthropometrics. Second, to increase the comparability between studies and to facilitate the assessment of effectiveness, more agreement is needed for best measures of the diet and questionnaires. Third, more research is needed to be done among specific groups like low socio-economic group, immigrants or minorities. As mention earlier, only few listed studies included this specific group in their studies. Furthermore, evidence suggest that health inequalities such as prevalence of overweight are as a result of dietary habits and ethnicity and socio-economic status are identified as determinants of health eating. Thus, future research should not exclude these specific groups as European countries have become ethnically diverse.

To improve or decrease childhood diseases such as overweight and obesity and other aspects of health, many policy documents have been calling for the development of the effective strategies among children’s and adolescents. Even though the limited to moderate impact and evidence was found among these school-based interventions, it should be noted that interventions were not primarily targeting obesity prevention but, in many cases, had a broader scope. Thus, in order to deliver these evidence-based recommendations to policy makers factors such as sustainability of intervention, context and cost effectiveness should be considered. Additionally, the policy makers should ensure school policies and the environment that encourage physical activity and a healthy diet.

## 5. Conclusions

Findings from this systematised review suggest that applying multicomponent interventions (environmental, educational, and physical strategies) along with parental involvement and of long-term initiatives may be promising for improving dietary habits and other childhood related diseases among primary school children. Despite being challenging to find experimental studies done in related fields, those studies found showed positive trend. Thus, to conclude, evidence of the effect was found among school-based food and nutrition initiatives among primary school children. However, to strengthen the perspectives of this study, further systematic review targeting the more long-term studies assessing the long-term sustainability of the interventions should be considered. Also, studies with goal to increase efficiency of anthropometric measurements in their future school-based interventions could include increasing PA, increasing fruit and vegetable intake and decreasing sedentary behaviour. This study has provided fundamentals background on which further research could be done in this area of school-based food and nutrition interventions. Thus, the findings from this systematic review can be used as guidelines for future interventions in school settings related to food and nutrition. Also, the categorization of intervention components we see as useful for the planning of future interventions.

## Figures and Tables

**Table 1:** Coding table for study designs. The table shows the types of studies examined in the review and the power assigned to them.

Code	Design	Power
PP	Pre-Test/Post-Test	1
OBS	Observational	1
CT	Controlled Trial	2
RCT	Randomized Controlled Trial	3
RCCT	Randomized Controlled Cluster Trial	3

**Table 2:** The review sample: study design/characteristics. The table shows the 43 studies of the review Illustrating study design and study characteristics of the included studies.

Author	Year	Title/Reference	Main Aim (from Abstract)	Main Aim in Brief	Program Name	Location & Country	Study Design	Study Design Coded	Power	Intervention Components			
					Acronym		Column I	RCT, PP, CT, RCCT, Quasi		Information and Teaching	Food Focus		Family/Social Support
											Environmental/Food Focus on Healthy Meal Availability	Environmental/Food Focus through School Gardening	
Harake et al. [35]	2018	Impact of pilot school-based nutrition intervention on dietary knowledge, attitudes, behaviours and nutritional status of Syrian refugee children in the Bekaa, Lebanon	This study aimed to evaluate the impact of a six-month pilot school-based nutrition intervention on changes in dietary knowledge, attitude, and behavior of Syrian refugee children enrolled in informal primary schools located in the rural region of the Bekaa in Lebanon. A secondary objective of the study was to explore the effect of the intervention on the dietary intake and nutritional status of children.	Nutritional knowledge, attitude, HE & FV	GHATA	Bekaa Lebanon	Quasi experimental	QED	1	x		x	
Adab P, et al. [36]	2018	Effectiveness of a childhood obesity prevention programme delivered through schools, targeting >6 (more than 6 years) and 7 years old cluster randomised controlled trial (WAVES study)	To assess the effectiveness of a school and family based healthy lifestyle programme (WAVES intervention) compare with usual practice, in preventing childhood obesity.	Anthropometry, HE & FV	WAVES	UK primary schools from the West Midlands within 35 miles of the study centre	Randomized Controlled Cluster Trials	RCCT	3	x			
Harley A, et al. [37]	2018	Youth Chef Academy: Pilot Results From a Plant-Based Culinary and Nutrition Literacy Program for Sixth and Seventh Graders	The study aim was to examine the effectiveness of Youth Chef Academy (YCA), a classroom-based experiential culinary and nutrition literacy intervention for sixth and seventh graders (11- to 13-year-old) designed to impact healthy eating.	HE & FV, Nutritional knowledge	YCA	US (exact location is missing)	Controlled Trial (CT)	CT	2	x			
Hermans R.C.J. et al. [38]	2018	Feed the Alien! The Effects of a Nutrition Instruction Game on Children’s Nutritional Knowledge and Food Intake	The aim of this study was to test the short-term effectiveness of the Alien Health Game, a videogame designed to teach elementary school children about nutrition and healthy food choices.	HE & FV, Nutritional knowledge	AHG	Dutch, Netherland	Pre-test post-test, experimental study design	QED	1	x			
Piana N., et al. [39]	2017	An innovative school-based intervention to promote healthy lifestyles	To describe an innovative school-based intervention to promote healthy lifestyles. To evaluate its effects on children’s food habits and to highlight the key components which contribute most to the beneficial effects obtained from children’s, teachers’ and parents’ perspectives.	HE & FV, Nutritional knowledge, Physical activity	Kidmed test	Spoleto, Umbria	Pre-test post-test	PP	1	x			x
Battjes-Fries M.C.E., et al. [40]	2017	Effectiveness of Taste Lessons with and without additional experiential learning activities on children’s willingness to taste vegetables	The aim of this study was to assess the effect of Taste Lessons with and without extra experiential learning activities on children’s willingness to taste unfamiliar vegetables, food neophobia, and vegetable consumption.	HE & FV, attitude	TLVM	Dutch province of Gelderland	Quasi experimental design	QED	1	x			
Bogart L.M., et al. [41]	2014	A Randomized Controlled Trial of Students for Nutrition and eXercise (SNaX): A Community-Based Participatory Research Study	To conduct a randomized controlled trial of Students for Nutrition and eXercise (SNaX), a 5-week middle-school-based obesity-prevention intervention combining school-wide environmental changes, multimedia, encouragement to eat healthy school cafeteria foods, and peer-led education.	HE & FV, Nutritional knowledge	SNaX	Los Angeles Unified School District	Randomized Controlled Trial	RCT	3	x			
Shriqui V.K., et al. [42]	2016	Effects of a School-Based Intervention on Nutritional Knowledge and Habits of Low-Socioeconomic School Children in Israel: A Cluster Randomized Controlled Trial	Examining the effect of a school-based comprehensive intervention on nutrition knowledge, eating habits, and behaviours among low socioeconomic status (LSES) school-aged children was performed	Anthropometry, HE & FV, Nutritional knowledge	NRI & PA	Beer Sheva, a big metropolis in southern Israel	Randomized Controlled Cluster Trial	RCCT	3	x			x
Sharma S.V. et al. [43]	2016	Evaluating a school-based fruit and vegetable co-op in low-income children: A quasi-experimental study	The purpose of this study was to evaluate the effectiveness of a new school-based food co-op program, Brighter Bites (BB), to increase fruit and vegetable intake, and home nutrition environment among low-income 1st graders and their parents.	HE & FV, Nutritional knowledge	BB	Houston, Texas	Quasi-experimental non-randomized controlled study	QED	1	x		x	x
Lawlor A.D. et al. [44]	2016	The Active for Life Year 5 (AFLY5) school-based cluster randomised controlled trial: effect on potential mediators	To determine the effect of the intervention on potential mediators	Anthropometry, HE & FV	AFLY5	South East of England	Cluster RCT	RCCT	3	x			x
Steyn P.N. et al. [45]	2016	Did Health kick, a randomised controlled trial primary school nutrition intervention improve dietary quality of children in low-income settings in South Africa?	To promote healthy eating habits and regular physical activity in learners, parents and educators by means of an action planning process	HE & FV, PA	HK	Western Cape (WC) Province	Cluster RCT	RCCT	3	x			
Jones M. et al. [46]	2017	Association between Food for Life, a Whole Setting Healthy and Sustinable Food Programme, and Primary School Children’s Consumption of Fruit and Vegetables: A cross Sectional Study in England	The aim of the study was to examine the association between primary school engagement in the Food for Life programme and the consumption of fruit and vegetables by children aged 8–10 years.	HE & FV, Nutritional knowledge	FLP	England	Cross sectional school matched comparison approach	Cross-sectional study design	1	x		x	
Larsen L.A. et al. [47]	2015	RE-AIM analysis of a randomized school-based nutrition intervention among fourth-grade classrooms in California	To promote healthy eating behaviours and attitudes in children	HE & FV, Nutritional knowledge, Attitude	NPP	California	RCT with pre-, post-, and follow-up assessments	RCT	3	x			x
Shen, Hu and Sun [48]	2015	Assessment of School-Based Quasi-Experimental Nutrition and Food Safety Health Education for Primary School Students in Two Poverty-Stricken Counties of West China	Aimed to assess the reliability of the knowledge, attitude and behaviour of nutrition and food safety questionnaire for primary school students (Grade 4 to 6) in poverty-stricken counties of China, and evaluate the effectiveness of health education through a quasi experiment, in order to promote policy establishment for child and adolescent health in the future	HE & FV, Nutritional knowledge, Attitude	NFSE	West China (Shaanxi and Yunnan provinces)	Quasi-experimental design	QED	1	x			
Gallotta C.M. et al. [21]	2016	Effects of combined physical education and nutritional programs on schoolchildren’s healthy habits	To evaluate the efficacy of three 5-month combined physical education (PE) and nutritional interventions on body composition, physical activity (PA) level, sedentary time and eating habits of schoolchildren	Anthropometry, HE & FV, Nutritional knowledge, PA	ESFS	Rome (Italy)	Randomised Controlled Cluster Trial	RCCT	3	x			x
Fairclough J.S. et al. [49]	2013	Promoting healthy weight in primary school children through physical activity and nutrition education: a pragmatic evaluation of the CHANGE! randomised intervention study	To assess the effectiveness of the CHANGE! intervention on measures of body size, PA and food intake	Anthropometry, HE & FV, PA	CHANGE	Wigan Borough in northwest England, UK	Cluster randomised intervention	RCCT	3	x			
Cunha B.D. et al. [50]	2013	Effectiveness of a randomized school-based intervention involving families and teachers to prevent excessive weight gain among Adolescents in Brazil	To evaluate the effectiveness of a school-based intervention involving the families and teachers that aimed to promote healthy eating habits in adolescents; the ultimate aim of the intervention was to reduce the increase in body mass index (BMI) of the students	Anthropometry, HE & FV, PA	PAPPAS	Duque de Caxias, Rio de Janeiro, Brazil	Paired cluster randomized school-based trial	RCCT	3	x			
Aviles O.A. et al. [51]	2017	A school-based intervention improved dietary intake outcomes and reduced waist circumference in adolescents: a cluster randomized controlled trial	The program aimed at improving the nutritional value of dietary intake, physical activity (primary outcomes), body mass index, waist circumference and blood pressure (secondary outcomes)	Anthropometry, HE & FV, PA	ACTIVITAL	Urban area of Cuenca, Ecuador	Pair-matched cluster randomized controlled trial	RCCT	3	x			x
Muros J.J. et al. [52]	2013	Results of a seven-week school-based physical activity and nutrition pilot program on health-related parameters in primary school children in Southern Spain	To determine the effect of nutrition education combined with sessions of vigorous extracurricular physical activity (VEPA) on the improvement of health-related parameters in children in primary education	Anthropometry, HE & FV, PA	VEPA	Southern Spain	Pilot study, PP	QED	1	x			
Moss A et al. [53]	2013	Farm to School and Nutrition Education: Positively Affecting Elementary School-Aged Children’s Nutrition Knowledge and Consumption Behavior	To introduce the CATCH nutrition curriculum and Farm to School program to assess nutrition knowledge of 3rd grade students, and increase their fruit and vegetable consumption behavior	HE & FV, Nutritional knowledge	CATCH	Southern Illinois	Quasi-experimental design	QED	1	x			
Zota D. et al. [54]	2016	Promotion of healthy nutrition among students participating in a school food aid program: a randomized trial	To evaluate the potential benefits on students’ eating habits, of incorporating healthy nutrition education as part of a school food aid program	Anthropometry, HE & FV	DIATROFI	Greece	Randomised Controlled Trial with the aspects of pre and post intervention questionnarie	RCT	3	x		x	x
Gold A. et al. [55]	2017	Classroom Nutrition Education Combined With Fruit and Vegetable Taste Testing Improves Children’s Dietary Intake	To test the classroom curriculum, go wild with fruits & veggies! (GWWFV) effectiveness to increase FV intake of third graders in rural and urban communities in North Dakota	HE & VF	GWWFV	North Dakota	Intervention study with RCT aspects (the schools were randomized to control and intervention school)	RCT, Intervention study	3	x			
Mbhatsani H.V., et al. [56]	2017	Development and Implementation of Nutrition Education on Dietary Diversification for Primary School Children	To ensure that people consume a variety of foods that, together, provide adequate quantities of all the essential micronutrients necessary for health	HE & FV, Nutritional knowledge	NET & HBoIF	Vhembe District of Limpopo Province in South Africa	Quasi-experimental, with a one-group pre-test/post-test intervention	QED	1	x			
Hutchinson J. et al. [57]	2015	Evaluation of the impact of school gardening interventions on children’s knowledge of and attitudes towards fruit and vegetables. A cluster randomised controlled trial	To evaluate whether ongoing gardening advice and gardening involvement from the Royal Horticultural Society (RHS) gardening specialists was associated with better fruit and vegetable outcomes in children than those at teacherled schools that obtained standard advice from the RHS Campaign for School Gardening	Nutritional knowledge, Attitude	CFSG	London boroughs, Wandsworth, Tower Hamlets, Greenwich and Sutton	Randomised Controlled Cluster Trial	RCCT	3	x	x		
Viggiano A et al. [58]	2018	Healthy lifestyle promotion in primary schools through the board game Kaledo: a pilot cluster randomized trial	The board game Kaledo seems to improve knowledge in nutrition and helps to promote a healthy lifestyle in children attending middle and high schools. So, this study was conducted to investigate whether similar effects of Kaledo could be found in younger children in primary school.	Anthropometry, HE & FV, Nutritional knowledge	Kaledo	Campania, Italy	Pilot cluster randomized trial	RCCT	3	x			
Waters E. et al. [59]	2017	Cluster randomised trial of a school-community child health promotion and obesity prevention intervention: findings from the evaluation of fun ‘n healthy in Moreland!	Fun ‘n healthy in Moreland! aimed to improve child adiposity, school policies and environments, parent engagement, health behaviours and child wellbeing	Anthropometry, HE & FV	FHM	Victoria, Australia	Randomised Controlled Cluster Trial	RCCT	3	x			
Xu F et al. [60]	2015	Effectiveness of a Randomized Controlled Lifestyle Intervention to Prevent Obesity among Chinese Primary School Students: CLICK-Obesity Study	To evaluate whether the lifestyle intervention was able to reduce obesity risk and increase healthy behaviors and knowledge	Anthropometry, Nutritional knowledge	CLICK-Obesity	Mainland China	Randomised Controlled Cluster Trial	RCCT	3	x			x
Jung et al. [61]	2018	Influence of school-based nutrition education program on healthy eating literacy and healthy food choice among primary school children	To examine the effectiveness of a school-based healthy eating intervention program, the Healthy Highway Program, for improving healthy eating knowledge and healthy food choice behavior among elementary school students	Nutritional knowledge, HE & FV	Healthy highway program	Oswego County, New York State	Pre-/post-test	QED	1	x			
Jhou W et al. [62]	2014	Effectiveness of a school-based nutrition and food safety education program among primary and junior high school students in Chongqing, China	To examine the effectiveness of a school-based nutrition and food safety education program among primary and junior high school students in China	Nutritional knowledge, attitude	school-based nutrition and food safety education	Chongqing, China	Pre-/post-test	QED	1	x			
Anderson EL, et al. [63]	2016	Long-term effects of the Active for Life Year 5 (AFLY5) school-based cluster-randomised controlled trial	To investigate the long-term effectiveness of a school-based intervention to improve physical activity and diet in children.	HE & FV, PA	AFLY5	Southwest of England	Randomised Controlled Cluster Trial	RCCT	3	x			
Griffin T.L. et al. [64]	2015	A Brief Educational Intervention Increases Knowledge of the Sugar Content of Foods and Drinks but Does Not Decrease Intakes in Scottish Children Aged 10–12 Years	To assess the effectiveness of an educational intervention to improve children’s knowledge of the sugar content of food and beverages	Nutritional knowledge, attitude	NEMS	Aberdeen, Scotland	Randomised Controlled Cluster Trial	RCCT	3	x			
Kipping R.R. et al. [65]	2014	Effect of intervention aimed at increasing physical activity, reducing sedentary behaviour, and increasing fruit and vegetable consumption in children: Active for Life Year 5 (AFLY5) school-based cluster randomised controlled trial	To investigate the effectiveness of a school-based intervention to increase physical activity, reduce sedentary behaviour, and increase fruit and vegetable consumption in children	HE & FV, PA	AFLY5	South west of England	Randomised Controlled Cluster Trial	RCCT	3	x			
Gaar V.M. et al. [66]	2014	Effects of an intervention aimed at reducing the intake of sugar-sweetened beverages in primary school children: a controlled trial	Aimed at reducing children’s SSB consumption by promoting the intake of water	Nutritional knowledge, attitude	Water campaign	Rotterdam, Netherland	Controlled trial	CT	2	x			
Moore GF et al. [67]	2014	Impacts of the Primary School Free Breakfast Initiative on socio-economic inequalities in breakfast consumption among 9–11-year-old schoolchildren in Wales	To examine the impacts of the Primary School Free Breakfast Initiative in Wales on inequalities in children’s dietary behaviours and cognitive functioning	HE & FV	FSM	Wales, UK	Randomised Controlled Cluster Trial	RCCT	3			x	
Nyberg G. et al. [68]	2016	Effectiveness of a universal parental support programme to promote health behaviours and prevent overweight and obesity in 6-year-old children in disadvantaged areas, the Healthy School Start Study II, a cluster-randomised controlled trial	To develop and evaluate the effectiveness of a parental support programme to promote healthy dietary and physical activity habits and to prevent overweight and obesity in six-year-old children in disadvantaged areas	Anthropometry, HE & FV	A Healthy School Start	Stockholm, Sweden	Randomised Controlled Cluster Trial	RCCT	3	x			
Mittmann S., Austel A., and Ellrott T. [69]	2016	Behavioural effects of a short school-based fruit and vegetable promotion programme: 5-a-Day for kids	To evaluate the acceptance of the scheme as well as the short- and intermediate-term effects of the German “5-a-day for kids” project	HE & FV	5-a-day for kids	Hannover, Germany	Pre-/post-test	PP	1	x		x	
Huys N. et al. [70]	2019	Effect and process evaluation of a real-world school garden program on vegetable consumption and its determinants in primary schoolchildren	To investigate the effectiveness of a school garden program on children’s vegetable consumption and determinants and to gain insight into the process of the program	HE & FV, Nutritional knowledge	Taste Garden	Ghent, Belgium	Non-equivalent pre-test. Post–test control group design	PP	1	x	x		
Weber K.S. et al. [71]	2017	Positive effects of promoting physical activity and balanced diet in a primary school setting with a high proportion of migrant school children	To evaluate the effects of a school-based intervention offering additional hours of supervised physical activity and dietary education for 3rd and 4th graders in primary schools	HE & FV, Nutritional knowledge	‘Be smart. Join in. Be fit.’	Düsseldorf, Germany	Controlled trial	CT	2	x			
Llargue’s E. et al. [72]	2016	Four-year outcomes of an educational intervention in healthy habits in schoolchildren: the Avall 3 Trial	To investigate the impact of the intervention on physical activity, BMI and prevalence of overweight and obesity after 4 years	Anthropometry	The Avall project	Granollers, Spain	Randomised Controlled Cluster Trial	RCCT	3	x			
Martins M.L. et al. [73]	2015	Strategies to reduce plate waste in primary schools—experimental evaluation	To determine and compare the effect of two interventions in reducing the plate waste of school lunches	Nutritional Knowledge	Reduce plate waste	City of Porto, Portugal	Controlled trial	CT	2	x		x	
Rosario R. et al. [74]	2016	Impact of a school-based intervention to promote fruit intake: a cluster randomized controlled trial	To examine the effects of a six-month dietary education intervention programme, delivered and taught by trained teachers, on the consumption of fruit as a dessert in children aged 6–12 years	HE & FV	Dietary education intervention programme	City in north of Portugal	Randomised Controlled Cluser Trial	RCCT	3	x			
Zafiropulos V. et al. [75]	2015	Preliminary results of a dietary intervention among primary school children	To evaluate the effectiveness of the dietary intervention by measuring body composition and dietary behavior of children prior to and after the intervention	Anthropometry, HE & FV	WBDI	central/eastern Crete Greece	RCT with the aspects of pre and post intervention	RCT	3	x			

**Table 3:** The review sample-findings. The table shows the findings from the 43 studies of the review.

Author	Year	Age	Age Coded			Sample Size, n	Time Duration/Month	Outcome Measures				Effectiveness Among Children				Target Group	Target Group Coded	
		Years	EA	EML	EL			Anthropometry	HE/FV	Nutritional Knowledge	Attitude	Anthropometry	HE/FV	Nutritional Knowledge	Attitude		S	NS
Harake et al. [35]	2018	6–14 years	x	x	x	183	6	x	x	x	x	3	3	4	2	Syrian refugee children in grade 4 to 6 from three informal primary schools (2 intervention and one control)		x
Adab P, et al. [36]	2018	6–7 years	x			1392	12	x	x			1	1			UK primary schools	x	
Harley A, et al. [37]	2018	11–13 years			x	248	1 and half		x	x			4	4		8 public kindergarten		x
Hermans R.C.J. et al. [38]	2018	10–13 years		x	x	108	N.A.		x	x			1	1		Dutch children (elementary school children)—3 primary school in the souther part of Netherland	x	
Piana N., et al. [39]	2017	7–9 years	x	x		190	4		x	x			4	4		11 primary school classes in five schools	x	
Battjes-Fries M.C.E., et al. [40]	2017	10–11 years		x	x	1010	3		X		X		1		1	children of 34 elementary school grade 6 and 7	x	
Bogart L.M., et al. [41]	2014	N.A.				2997	41		x	x			4	4		10 schools	x	
Shriqui V.K., et al. [42]	2016	4–7 years	x			240	10	x	x	x		2	4	4		Children attending LSES school classes	x	
Sharma S.V. et al. [43]	2016	N.A. (first grade students)	x			172	24		x	x			3	3		Public or charter schools 1st grade students and their family members		x
Lawlor A.D. et al. [44]	2016	9–10 years		x		2221 (valid data for the 10 mediators were available for 87% to 96% of participants	36	x	x			1	1			primary school children	x	
Steyn P.N. et al. [45]	2016	Mean age 9.9 years	x			500 intervention and 498 control	36		x				1			primary school children from low income settings	x	
Jones M. et al. [46]	2017	8–10 years		x		2411	24		x	x			4	4		schools engaged with the Food for Life programme	x	
Larsen L.A. et al. [47]	2015	(fourth grade students) average 9 years	x			1713	2		x	x	x		4	4	3	47 fourth-grade California classrooms	x	
Shen, Hu and Sun [48]	2015	10.80 ± 1.14		x		478	8		x	x	x		4	4	1	Twelve primary schools in west China	x	
Gallotta C.M. et al. [21]	2016	8–11 years		x	x	230	5	x	x	x		3	4	3		three primary schools in the rural area in the north of the city of Rome (Italy)	x	
Fairclough J.S. et al. [49]	2013	10–11 years		x	x	318	6	x	x			3	1			12 primary schools	x	
Cunha B.D. et al. [50]	2013	10–11 years		x	x	574	9	x		x		1		3		20 schools with fifth grade classes	x	
Aviles O.A. et al. [51]	2017	12–14 years			x	1430	28	x	x			2	3			20 schools	x	
Muros J.J. et al. [52]	2013	10–11 years		x	x	54	2	x	x			2	2			2 schools from rular environment with same socio economic status	x	
Moss A et al. [53]	2013	N.A. 3rd grade students				65	1		x	x			3	4		3rd grade students	x	
Zota D. et al. [54]	2016	4–11 years	x	x	x	21261	12	x	x	x		3	4	3		students attending both elementary and secondary schools in areas of low socioeconomic status (SES)	x	
Gold A. et al. [55]	2017	8–9 years		x		662	12		x				4			3rd grade children from 26 schools	x	
Mbhatsani H.V., et al. [74]	2017	9–14 years		x	x	172	6		x	x			3	3		2 rural primary schools with similar socioeconomic backgrounds	x	
Hutchinson J. et al. [57]	2015	7–10 years	x	x		1256	12		x	x	x		3	3	2	21 London schools	x	
Viggiano A et al. [58]	2018	7–11 years	x	x	x	1313	2.5	x	x	x		2	3	3		10 primary schools	x	
Waters E. et al. [59]	2017	5–12 years	x	x	x	2965	42	x	x	x		1	3	3		24 schools of Moreland municipality	x	
Xu F et al. [60]	2015	Mean age 10.2		x		1182	10	x		x		2		3		4th grade students from 8 schools of Nanjing, China	x	
Jung et al. [61]	2018	NA (elementry school-kindergarden, 2nd, 3rd, 4th, 5th and 6th graders)	x	x	x	646	12		x	x	x		2	3	2	2 elementary schools	x	

**Table 4:** Descriptive statistics.

	*N*	Minimum	Maximum	Mean	Standard Deviation
Power	42	1.00	3.00	2.2619	0.91223
InfoAndTeach	42	0.00	1.00	0.9762	0.15430
FandV	42	0.00	1.00	0.0714	0.26066
FreeFood	42	0.00	1.00	0.0476	0.21554
AvailFood	42	0.00	1.00	0.1667	0.37720
FamilySocialSupport	42	0.00	1.00	0.2143	0.41530
EA—early age	42	0.00	1.00	0.3810	0.49151
EML—early middle late age	42	0.00	1.00	0.7381	0.44500
EL—early late age	42	0.00	1.00	0.5476	0.50376
SampleSize	42	54.00	21261.00	1464.2619	3277.18184
log10SampleSize	42	1.73	4.33	2.7904	0.54986
Months	41	1.00	112.00	14.6585	19.00245
YearOrMore01	41	0.00	1.00	0.4390	0.50243
AnthropometryScale	18	0.00	4.00	2.0000	1.13759
HEFVScale	36	1.00	4.00	2.5556	1.27491
NutritionalKnowledgeScale	26	1.00	4.00	3.1923	0.89529
AttitudeScale	9	1.00	3.00	1.7778	0.66667

**Table 5:** Linear regression model for attitude.

Model	Unstandardized Coefficients		Standardized Coefficients	t	Significance
	B	SE	Beta		
(Constant) FamilySocialSupport EA—early age	1.250	0.177		7.071	0.000
	1.000	0.395	0.500	2.530	0.045
	0.750	0.250	0.593	3.000	0.024

**Table 6:** Linear regression model for anthropometry.

Model	Unstandardized Coefficients		Standardized Coefficients	t	Significance
	B	SE	Beta		
(Constant) Power AvailFood YearOrMore log10SampleSize	4.140	1.008		4.109	0.001
	1.511	0.468	0.859	3.231	0.007
	3.432	0.804	0.976	4.267	0.001
	0.870	0.403	0.384	2.161	0.050
	−2.437	0.503	−1.267	−4.846	0.000

**Table 7:** Linear regression model for behaviour.

Model	Unstandardized Coefficients		Standardized Coefficients		t	Significance	
	B	SE	Beta				
(Constant) FamilySocialSupport	2.321	0.229		10.131	0.000	
	1.054	0.486	0.348	2.168	0.037		

**Table 8:** Alternative linear regression model for behaviour.

Model	Unstandardized Coefficients		Standardized Coefficients	t	Significance
	B	SE	Beta		
(Constant) Neurope	3.227	0.205		15.761	0.000
	−1.727	0.328	−0.670	−5.260	0.000

## References

[1] Micha R., Karageorgou D., Bakogianni I., Trichia E., Whitsel L.P., Story M., Penalvo J.L., Mozaffarian D. (2018). Effectiveness of school food environment policies on children’s dietary behaviors: A systematic review and meta-analysis. PLoS ONE.

[2] WHO Europe (2006). Food and Nutrition Policy for Schools Copenhagen, Security PfNaF.

[3] EU Commission (2014). EU Action Plan on Childhood Obesity 2014–2020.

[4] Lytle L.A., Kubik M.Y. (2003). Nutritional issues for adolescents. Best Pract. Res. Clin. Endocrinol. Metab..

[5] WHO Obesity and Overweight 2020. https://www.who.int/news-room/fact-sheets/detail/obesity-and-overweight.

[6] WHO (2003). Diet, Nutrition and The Prevention of Chronic Diseases.

[7] Serra-Paya N., Ensenyat A., Castro-Vinuales I., Real J., Sinfreu-Bergues X., Zapata A., Mur J.M., Galindo-Ortego G., Solé-Mir E., Teixido C. (2015). Effectiveness of a Multi-Component Intervention for Overweight and Obese Children (Nereu Program): A Randomized Controlled Trial. PLoS ONE.

[8] Hruby A., Hu F.B. (2015). The Epidemiology of Obesity: A Big Picture. Pharmacoeconomics.

[9] WHO (2015). Interim Report of the Commission on Ending Childhood Obesity.

[10] Aceves-Martins M., Llauradó E., Tarro L., Moreno-García C.F., Trujillo Escobar T.G., Sola R., Giralt M. (2016). Effectiveness of social marketing strategies to reduce youth obesity in European school-based interventions: A systematic review and meta-analysis. Nutr. Rev..

[11] Cali A.M.G., Caprio S. (2008). Obesity in children and adolescents. J. Clin. Endocrinol. Metab..

[12] Abdelaal M., le Roux C.W., Docherty N.G. (2017). Morbidity and mortality associated with obesity. Ann. Transl. Med..

[13] WHO Child and Adolescent Health Copenhagen: UN City 2019. http://www.euro.who.int/en/health-topics/Life-stages/child-and-adolescent-health/child-and-adolescent-health.

[14] Lynch C., Kristjansdottir A.G., Te Velde S.J., Lien N., Roos E., Thorsdottir I., Krawinkel M., de Almeida M.D.V., Papadaki A., Ribic C.H. (2014). Fruit and vegetable consumption in a sample of 11-year-old children in ten European countries—The PRO GREENS cross-sectional survey. Public Health Nutr..

[15] Rippin H.L., Hutchinson J., Jewell J., Breda J.J., Cade J.E. (2019). Child and adolescent nutrient intakes from current national dietary surveys of European populations. Nutr. Res. Rev..

[16] EU Science Hub Childhood Obesity: Local Data Feeds Local Solutions 2018. https://ec.europa.eu/jrc/en/news/childhood-obesity-local-data-feeds-local-solutions.

[17] Stefan S.G.B., Joao B., Sandra C., Michael N., Jan W. (2019). School Food and Nutrition in Europe: Policies, Interventions and Their Impact: EU Science Hub. https://ec.europa.eu/jrc/en/publication/eur-scientific-and-technical-research-reports/school-food-and-nutrition-europe-policies-interventions-and-their-impact.

[18] WHO (2018). Obesity UN City Marmorvej 51 DK-2100 Copenhagen Ø: WHO Regional Office for Europe. http://www.euro.who.int/en/health-topics/noncommunicable-diseases/obesity/obesity.

[19] ROfE WHO (2014). WHO European Childhood Obesity Surveillance Initiative (COSI): UN City.

[20] Gallotta M.C., Iazzoni S., Emerenziani G.P., Meucci M., Migliaccio S., Guidetti L., Baldari C. (2016). Effects of combined physical education and nutritional programs on schoolchildren’s healthy habits. PeerJ.

[21] Contento I.R. (2008). Nutrition education: Linking research, theory, and practice. Asia Pac. J. Clin. Nutr..

[22] Glanz K. (2009). Measuring Food Environments: A Historical Perspective. Am. J. Prev. Med..

[23] Vereecken C., Haerens L., De Bourdeaudhuij I., Maes L. (2010). The relationship between children’s home food environment and dietary patterns in childhood and adolescence. Public Health Nutr..

[24] Nutrition UNSSCo (2017). Schools as a System to Improve Nutrition.

[25] Hollar D., Messiah S.E., Lopez-Mitnik G., Hollar T.L., Almon M., Agatston A.S. (2010). Effect of a Two-Year Obesity Prevention Intervention on Percentile Changes in Body Mass Index and Academic Performance in Low-Income Elementary School Children. Am. J. Public Health.

[26] Florence M.D., Asbridge M., Veugelers P.J. (2008). Diet Quality and Academic Performance. J. Sch. Health.

[27] Anderson M.L., Gallagher J., Ritchie E.R. (2018). School meal quality and academic performance. J. Public Econ..

[28] Amare B., Moges B., Fantahun B., Tafess K., Woldeyohannes D., Yismaw G., Ayane T., Yabutani T., Mulu A., Ota F. (2012). Micronutrient levels and nutritional status of school children living in Northwest Ethiopia. Nutr. J..

[29] Herrador Z., Sordo L., Gadisa E., Buño A., Gómez-Rioja R., Iturzaeta J.M., Armas L.F.D., Benito A., Aseffa A., Moreno J. (2014). Micronutrient Deficiencies and Related Factors in School-Aged Children in Ethiopia: A Cross-Sectional Study in Libo Kemkem and Fogera Districts, Amhara Regional State. PLoS ONE.

[30] Christian P., Murray-Kolb L.E., Khatry S.K., Katz J., Schaefer B.A., Cole P.M., Leclerq S.C., Tielsch J.M. (2010). Prenatal Micronutrient Supplementation and Intellectual and Motor Function in Early School-aged Children in Nepal. JAMA.

[31] The NEMO Study Group (2007). Effect of a 12-mo micronutrient intervention on learning and memory in well-nourished and marginally nourished school-aged children: 2 parallel, randomized, placebo-controlled studies in Australia and Indonesia. Am. J. Clin. Nutr..

[32] Effective Public Health Practice Project. https://merst.ca/ephpp/.

[33] Van Cauwenberghe E., Maes L., Spittaels H., van Lenthe F.J., Brug J., Oppert J.M., De Bourdeaudhuij I. (2010). Effectiveness of school-based interventions in Europe to promote healthy nutrition in children and adolescents: Systematic review of published and ‘grey’ literature. Br. J. Nutr..

[34] El Harake M.D., Kharroubi S., Hamadeh S.K., Jomaa L. (2018). Impact of a Pilot School-Based Nutrition Intervention on Dietary Knowledge, Attitudes, Behavior and Nutritional Status of Syrian Refugee Children in the Bekaa, Lebanon. Nutrients.

[35] Adab P., Pallan M.J., Lancashire E.R., Hemming K., Frew E., Barrett T., Bhopal R., Cade J.E., Canaway A., Clarke J.L. (2018). Effectiveness of a childhood obesity prevention programme delivered through schools, targeting 6 and 7 year olds: Cluster randomised controlled trial (WAVES study). BMJ.

[36] Harley A., Lemke M., Brazauskas R., Carnegie N.B., Bokowy L., Kingery L. (2018). Youth Chef Academy: Pilot Results From a Plant-Based Culinary and Nutrition Literacy Program for Sixth and Seventh Graders. J. Sch. Health.

[37] Hermans R.C., van den Broek N., Nederkoorn C., Otten R., Ruiter E.L., Johnson-Glenberg M.C. (2018). Feed the Alien! The Effects of a Nutrition Instruction Game on Children’s Nutritional Knowledge and Food Intake. Games Health J..

[38] Piana N., Ranucci C., Buratta L., Foglia E., Fabi M., Novelli F., Casucci S., Reginato E., Pippi R., Aiello C. (2017). An innovative school-based intervention to promote healthy lifestyles. Health Educ. J..

[39] Battjes-Fries M.C., Haveman-Nies A., Zeinstra G.G., van Dongen E.J., Meester H.J., van den Top-Pullen R., van’t Veer P., de Graaf K. (2017). Effectiveness of Taste Lessons with and without additional experiential learning activities on children’s willingness to taste vegetables. Appetite.

[40] Bogart L.M., Cowgill B.O., Elliott M.N., Klein D.J., Dawson J.H., Uyeda K., Elijah J., Binkle D.G., Schuster M.A. (2014). A randomized controlled trial of students for nutrition and eXercise: A community-based participatory research study. J. Adolesc. Health.

[41] Kaufman-Shriqui V., Fraser D., Friger M., Geva D., Bilenko N., Vardi H., Elhadad N., Mor K., Feine Z., Shahar D.R. (2016). Effect of a School-Based Intervention on Nutritional Knowledge and Habits of Low-Socioeconomic School Children in Israel: A Cluster-Randomized Controlled Trial. Nutrients.

[42] Sharma S.V., Markham C., Chow J., Ranjit N., Pomeroy M., Raber M. (2016). Evaluating a school-based fruit and vegetable co-op in low-income children: A quasi-experimental study. Prev. Med..

[43] Lawlor D.A., Jago R., Noble S.M., Chittleborough C.R., Campbell R., Mytton J., Howe L.D., Peters T.J., Kipping R.R. (2016). The Active for Life Year 5 (AFLY5) school-based cluster randomised controlled trial: Effect on potential mediators. BMC Public Health.

[44] Steyn N.P., Villiers A.D., Gwebushe N., Draper C.E., Hill J., Waal M.D., Dalais L., Abrahams Z., Lombard C., Lambert E.V. (2015). Did HealthKick, a randomised controlled trial primary school nutrition intervention improve dietary quality of children in low-income settings in South Africa?. BMC Public Health.

[45] Jones M., Pitt H., Oxford L., Bray I., Kimberlee R., Orme J. (2017). Association between Food for Life, a Whole Setting Healthy and Sustainable Food Programme, and Primary School Children’s Consumption of Fruit and Vegetables: A Cross-Sectional Study in England. Int. J. Environ. Res. Public Health.

[46] Larsen A.L., Robertson T., Dunton G. (2015). RE-AIM analysis of a randomized school-based nutrition intervention among fourth-grade classrooms in California. Transl. Behav. Med..

[47] Shen M., Hu M., Sun Z. (2015). Assessment of School-Based Quasi-Experimental Nutrition and Food Safety Health Education for Primary School Students in Two Poverty-Stricken Counties of West China. PLoS ONE.

[48] Fairclough S.J., Hackett A.F., Davies I.G., Gobbi R., Mackintosh K.A., Warburton G.L., Stratton G., van Sluijs E.M., Boddy L.M. (2013). Promoting healthy weight in primary school children through physical activity and nutrition education: A pragmatic evaluation of the CHANGE! randomised intervention study. BMC Public Health.

[49] Cunha D.B., de Souza B.d.S.N., Pereira R.A., Sichieri R. (2013). Effectiveness of a randomized school-based intervention involving families and teachers to prevent excessive weight gain among adolescents in Brazil. PLoS ONE.

[50] Ochoa-Avilés A., Verstraeten R., Huybregts L., Andrade S., Camp J.V., Donoso S., Ramirez P.L., Lachat C., Maes L., Kolsteren P. (2017). A school-based intervention improved dietary intake outcomes and reduced waist circumference in adolescents: A cluster randomized controlled trial. Nutr. J..

[51] Muros J.J., Zabala M., Oliveras-López M.J., Ocaña-Lara F.A., de la Serra H.L.G. (2013). Results of a 7-Week School-Based Physical Activity and Nutrition Pilot Program on Health-Related Parameters in Primary School Children in Southern Spain. Pediatr. Exerc. Sci..

[52] Moss A., Smith S., Null D., Long R.S., Tragoudas U. (2013). Farm to School and Nutrition Education: Positively Affecting Elementary School-Aged Children’s Nutrition Knowledge and Consumption Behavior. Child Obes..

[53] Zota D., Dalma A., Petralias A., Lykou A., Kastorini C.M., Yannakoulia M., Karnaki P., Belogianni K., Veloudaki A., Riza E. (2016). Promotion of healthy nutrition among students participating in a school food aid program: A randomized trial. Int. J. Public Health.

[54] Gold A., Larson M., Tucker J., Strang M. (2017). Classroom Nutrition Education Combined With Fruit and Vegetable Taste Testing Improves Children’s Dietary Intake. J. Sch. Health.

[55] Mbhatsani V.H., Mbhenyane X.G., Mabapa S.N. (2017). Development and Implementation of Nutrition Education on Dietary Diversification for Primary School Children. Ecol. Food Nutr..

[56] Hutchinson J., Christian M.S., Evans C.E.L., Nykjaer C., Hancock N., Cade J.E. (2015). Evaluation of the impact of school gardening interventions on children’s knowledge of and attitudes towards fruit and vegetables. A cluster randomised controlled trial. Appetite.

[57] Viggiano E., Viggiano A., Costanzo A.D., Viggiano A., Viggiano A., Andreozzi E., Romano V., Vicidomini C., Tuoro D.D., Gargano G. (2018). Healthy lifestyle promotion in primary schools through the board game Kaledo: A pilot cluster randomized trial. Eur. J. Pediatr..

[58] Waters E., Gibbs L., Tadic M., Ukoumunne O.C., Magarey A., Okely A.D., Silva A.D., Armit C., Green J., O’Connor T. (2017). Cluster randomised trial of a school-community child health promotion and obesity prevention intervention: Findings from the evaluation of fun ‘n healthy in Moreland!. BMC Public Health.

[59] Xu F., Ware R.S., Leslie E., Tse L.A., Wang Z., Li J., Wang Y. (2015). Effectiveness of a Randomized Controlled Lifestyle Intervention to Prevent Obesity among Chinese Primary School Students: CLICK-Obesity Study. PLoS ONE.

[60] Jung T., Huang J., Eagan L., Oldenburg D. (2019). Influence of school-based nutrition education program on healthy eating literacy and healthy food choice among primary school children. Int. J. Health Promot. Educ..

[61] Zhou W.-J., Xu X., Li G., Sharma M., Qie Y.-L., Zhao Y. (2014). Effectiveness of a school-based nutrition and food safety education program among primary and junior high school students in Chongqing, China. Glob. Health Promot..

[62] Anderson E.L., Howe L.D., Kipping R.R., Campbell R., Jago R., Noble S.M., Wells S., Chittleborough C., Peters T.J., Lawlor D.A. (2016). Long-term effects of the Active for Life Year 5 (AFLY5) school-based cluster-randomised controlled trial. BMJ Open.

[63] Griffin T.L., Jackson D.M., McNeill G., Aucott L.S., Macdiarmid J.I. (2015). A Brief Educational Intervention Increases Knowledge of the Sugar Content of Foods and Drinks but Does Not Decrease Intakes in Scottish Children Aged 10–12 Years. J. Nutr. Educ. Behav..

[64] Kipping R.R., Howe L.D., Jago R., Campbell R., Wells S., Chittleborough C.R., Mytton J., Noble S.M., Peters T.J., Lawlor D.A. (2014). Effect of intervention aimed at increasing physical activity, reducing sedentary behaviour, and increasing fruit and vegetable consumption in children: Active for Life Year 5 (AFLY5) school based cluster randomised controlled trial. BMJ Br. Med J..

[65] Van de Gaar V.M., Jansen W., Grieken A.V., Borsboom G.J.J.M., Kremers S., Raat H. (2014). Effects of an intervention aimed at reducing the intake of sugar-sweetened beverages in primary school children: A controlled trial. Int. J. Behav. Nutr. Phys. Act..

[66] Moore G.F., Murphy S., Chaplin K., Lyons R.A., Atkinson M., Moore L. (2014). Impacts of the Primary School Free Breakfast Initiative on socio-economic inequalities in breakfast consumption among 9-11-year-old schoolchildren in Wales. Public Health Nutr..

[67] Nyberg G., Norman Å., Sundblom E., Zeebari Z., Elinder L.S. (2016). Effectiveness of a universal parental support programme to promote health behaviours and prevent overweight and obesity in 6-year-old children in disadvantaged areas, the Healthy School Start Study II, a cluster-randomised controlled trial. Int. J. Behav. Nutr. Phys. Act..

[68] Mittmann S., Austel A., Ellrott T. (2016). Behavioural effects of a short school-based fruit and vegetable promotion programme: 5-a-Day for kids. Health Educ..

[69] Huys N., Cardon G., Craemer D.M., Hermans N., Renard S., Roesbeke M., Stevens W., Lepeleere S.D., Deforche B. (2019). Effect and process evaluation of a real-world school garden program on vegetable consumption and its determinants in primary schoolchildren. PLoS ONE.

[70] Weber K.S., Spörkel O., Mertens M., Freese A., Strassburger K., Kemper B., Bachmann C., Diehlmann K., Stemper T., Buyken A.E. (2017). Positive Effects of Promoting Physical Activity and Balanced Diets in a Primary School Setting with a High Proportion of Migrant School Children. Exp. Clin. Endocrinol. Diabetes.

[71] Llargués E., Recasens M.A., Manresa J.-M., Jensen B.B., Franco R., Nadal A., Vila M., Recasens I., Perez M.J., Castel C. (2016). Four-year outcomes of an educational intervention in healthy habits in schoolchildren: The Avall 3 Trial. Eur. J. Public Health.

[72] Liz Martins M., Rodrigues S.S.P., Cunha L.M., Rocha A. (2016). Strategies to reduce plate waste in primary schools–experimental evaluation. Public Health Nutr..

[73] Rosário R., Araújo A., Padrão P., Lopes O., Moreira A., Abreu S., Vale S., Pereira B., Moreira P. (2016). Impact of a school-based intervention to promote fruit intake: A cluster randomized controlled trial. Public Health.

[74] Zafiropulos V., Chatzi V., Petros D., Markaki A., Zacharias F., Nikolaos T. Preliminary Results of A Dietary Intervention among Primary-School Children. Proceedings of the European Congress on Obesity.

[75] Charlebois J., Gowrinathan Y., Waddell P. (2012). A Review of the Evidence: School-based Interventions to Address Obesity Prevention in Children 6–12 Years of Age. Tor. Public Health.

[76] Delgado-Noguera M., Tort S., Martínez-Zapata M.J., Bonfill X. (2011). Primary school interventions to promote fruit and vegetable consumption: A systematic review and meta-analysis. Prev. Med..

[77] Evans C.E., Christian M.S., Cleghorn C.L., Greenwood D.C., Cade J.E. (2012). Systematic review and meta-analysis of school-based interventions to improve daily fruit and vegetable intake in children aged 5 to 12 y. Am. J. Clin. Nutr..

[78] Brown E.C., Buchan D.S., Baker J.S., Wyatt F.B., Bocalini D.S., Kilgore L. (2016). A Systematised Review of Primary School Whole Class Child Obesity Interventions: Effectiveness, Characteristics, and Strategies. BioMed Res. Int..

[79] Mikkelsen B.E., Kngesveen K., Afflerbach T., Barnekow V. (2016). The human rights framework, the school and healthier eating among young people a European perspective. Public Health Nutr..

[80] Rashid V., Engberink M.F., Eijsden M.V., Nicolaou M., Dekker L.H., Verhoeff A.P., Weijs P.J.M. (2018). Ethnicity and socioeconomic status are related to dietary patterns at age 5 in the Amsterdam born children and their development (ABCD) cohort. BMC Public Health.

